# Factors influencing seniors' willingness to pay intention for exercise in the civil sports and recreation centers

**DOI:** 10.3389/fpubh.2022.992500

**Published:** 2023-01-27

**Authors:** Chin-Yi Fang, Po-Yu Chen, Yung Liao

**Affiliations:** Graduate Institute of Sport, Leisure and Hospitality Management, National Taiwan Normal University, Taipei, Taiwan

**Keywords:** civil sports and recreation centers (CSRC), WTP more, exercise involvement, motivation, constraint

## Abstract

**Background:**

The increasing trend for an older population is a phenomenon that is well recognized around the world. The percentage of senior citizens participating in sports, recreation, and leisure activities is also increasing in order to sustain a healthier society. As long as seniors recognize the importance and benefits of keeping healthy, they would prefer to be active in various sports and fitness activities. The seniors could engage in making decisions about their own health by exercising regularly and by encouraging their similar-age peers. The civil sports and recreation centers (CSRCs) in Taiwan have adopted operating transfer (OT) and are located mainly in metropolitan areas, with programs consisting of indoor sports offering people daily exercise. The major aims of the CSRC are to keep the citizens in good health and not to be the city government's financial burden. The extant literature examines the determinants of general consumer behavior, neglecting studies on older adults. This study aims to explore the factors influencing seniors' willingness to pay (WTP) more for exercise in CSRCs outside of free hours.

**Methods:**

This mixed-method study consists of quantitative and qualitative analyses. For the quantitative component, we invited five experts in the sports industry from the industry and academia to evaluate the validity of the questionnaire. A questionnaire consisting of motivation, exercise involvement, and constraint constructs in the quantitative method was administered to 193 older adults in CSRCs. The collected data were analyzed through descriptive statistics, *t*-test, one-way analysis of variance (ANOVA), and structural equation model (SEM).

**Results:**

Empirical results indicate that WTP in groups of people aged 75–79, 70–74, and 65–69 years is higher than in the group of people aged 85 years and above. Exercise involvement and motivation had more of a positive impact on seniors' WTP.

**Discussions:**

Developing a regular exercise habits and having social interaction in exercise courses drive female seniors to pay more for body health. The results of this study provide managers of CSRCs with decision-making recommendations: (1) initiate entry-level sports courses, such as quick chair exercise, to cultivate among seniors the habit of exercise; (2) motivate seniors to participate in sports with other seniors in order to enhance their social activity and raise their WTP more for sports consumption outside of free scheduled periods; and (3) recruit seniors as exercise coaches or personal trainers to pass their experiences with other people of their age. In addition to expanding seniors' social connections, it encourages peers to exercise. Managerial implications and future research are also discussed.

**Conclusion:**

This study contributes to exploring the determinants of seniors' WTP intention. Motivation and exercise involvement had a significantly positive impact on seniors' WTP intention.

## Introduction

The World Health Organization (1) indicated that the number of people aged 60 years and above was 1 billion in 2019 and this number will increase to 1.4 billion by 2030 and 2.1 billion by 2050. The increasing trend for an older population is a phenomenon that is well recognized around the world. Senior adults are the most inactive sector of the U.S. population (2). The research claimed that senior adults may possibly face more constraints on physical activity (PA), such as lack of time, interest, fear of falling, and injuries (3). This is mainly due to the chronic diseases associated with age, most particularly, physical disability and discomfort leading to health constraints. Senior adults of low socioeconomic status (4), women (3), and those with less disposable income (5) are at greater risk of physical inactivity. Senior adults also report constraints that are more perceptual in nature, including lack of time, lack of motivation, misconceptions about PA, fear of injury, and lack of skill (6). As of 2017, an estimated 962 million people aged 60 years or above comprise 13% of the global population. The population of people aged 60 years and above is growing at a rate of about 3% per year. According to definitions from the World Health Organization, when the proportion of society that is elderly (over 65 years old) reaches 7%, it is called an “aged society,” while 14% is an “old-age society.” If this figure is 20% or more, it is a “super-aged society” (7). According to the Ministry of the Interior, the ratio of the elderly population in Taiwan exceeded 14% in 2018, and it became an aged society. The elderly population in Taiwan has accelerated. As of January 2021, Taiwan's elderly population (over 65 years old) accounted for 16.2% of the total population, officially making it an old-age society (8).

The percentage of senior citizens participating in sports, recreation, and leisure activities is increasing (9, 10). Consumers aged 60 years and over actively participate in a variety of sports and fitness activities because they acknowledge the importance and benefits of keeping fit and healthy (11). Shank (12) revealed that participation in sports for people in sports facilities, in particular, is closely associated with the lifestyle preferences and personalities of consumers. It is, therefore, important and necessary to understand senior adult consumers' exercise consumption habits. Currently, there are several exercise activities and courses offered at the civil sports and recreation centers (CSRCs) in Taiwan. Taiwan's CSRCs were built using the city government's funding and then were operated and transferred (OT) to private firms within the contracted years in order to make their operating performance better and generate revenue for the local authority. Taiwan's CSRCs have two major objectives of revenue generation and exercise participation increase because of the OT management type (13). Hence, the major aims of the CSRC are to keep the citizens in good health and not to be a local authority's financial burden. Meanwhile, to increase the percentage of senior citizens participating in PA, the CSRCs in Taiwan have been regulated to offer the following free welfare hour zone for people aged 65 years and above (hereafter, seniors): (1) 8:00–10:00 a.m. for the major sports facilities (excluding basketball, squash, and roller blades) during the whole week; and (2) 2:00–4:00 p.m. from Monday to Friday (only for swimming pool and gym). All free welfare hour zones exclude public holidays and summer and winter breaks. Outside these free welfare hour zones, seniors can buy a discounted ticket—NT$50 (US$1 = NT$28) for the swimming pool (normal ticket price is NT$110) and NT$25/h for the gym (normal ticket price is NT$50/h). However, there were limited senior participants to purchase these discounted tickets leading to the fact that the CSRCs could not generate revenue from the seniors and the seniors would not be interested in extending their exercise time.

There are limited studies on the physical and psychological needs of senior customers. However, ticket sales outside of these free welfare hours are not as good as the other operating periods. Even though the CSRCs are required to offer free welfare hour zones for people aged 65 years or above, few seniors experience PA in the CSRCs in Taiwan. In contrast, the CSRCs also observed that some seniors prefer to enjoy non-crowded PA during non-free welfare hours rather than experiencing PA during free welfare hours. In particular, some seniors do not like the feeling of being looked down upon during free welfare hours. Some CSRCs have attempted to initiate marketing campaigns, such as offering promotional packages that extend discounted periods for seniors and launching a PA course only for seniors.

Willingness to pay (WTP) is consumers' perceived value of products or services, usually expressed in their local currency (14). WTP usually consists of whether or not and how much a consumer is willing to pay for something (15). In economics, WTP is regarded as a contingent valuation that represents the maximum cost that a consumer is willing to pay for an item and shows the value of that item to the consumer (15, 16). However, Pulido-Fernández and López-Sánchez (17) asserted that there is still no appropriate statistical model to estimate WTP. Wertenbroch and Skiera (18) pointed out that consumers' WTP assesses the demand for public and private goods to plan an optimal price model. Previous studies have found that many research methods can assess the price of a consumer's different conditions. Lee and Heo (16) suggested that the value of products or services can be used to assess the benefits or costs of environmental resources. Namkung and Jang (19) used one item (“How much more are you willing to pay for green practices at the restaurant?”) to measure consumers' WTP more for green practices in restaurants. Yooyen and Leerattanakorn (20) asked the respondents to choose the amount or percentage that they were willing to pay from a list of possible payments. Table 1 shows the most important literature on WTP.

**Table 1:** Major related extant research on WTP.

**References**	**Issue**	**Field**	**Measure method, unit**
Lee and Heo (15)	WTP for renewable energy	Renewable energy sources	CVM, WTP, KRW$
Wertenbroch and Skiera (16)	WTP for retailed products	Retailed product	BDM method, choice bracketing procedure ($0.00–$10.00 in increments of $0.25)
Hu et al. (21)	Green restaurant	Green restaurant	SEM, green restaurant patronage (Survey)
	Patronage		
Namkung and Jang (19)	WTP more for green practices at restaurants	Green products	WTP more, $
Yooyen and Leerattanakorn (20)	WTP for organic pork	Agricultural products	Pay extra price, $
Ha-Brookshire and Norum (22)	WTP for socially responsible products—Cotton apparel	Agricultural products	Pay extra price, $
Pulido-Fernández and López-Sánchez (17)	WTP for sustainable destination in Spain.	Economic implications for sustainable tourists.	WTP more, $

CVM, contingent valuation method; BDM, Becker et al. (23) method.

There is no consensus among researchers about the best measurement for WTP (17). According to the results of Table 1, the survey questionnaire to estimate WTP more might be appropriate based on the previous studies (17, 19).

Exercise has been linked to improved physical health (24, 25). For senior adults, especially, exercise has been linked to better breathing, muscle strength, flexibility, and balance, less susceptibility to falls, and improved executive control processes (26–31). Motivation is defined as promoting and continuing activity programs in both the intensity and direction of the effort (32). People are motivated to exercise for personal goals and social interaction (33–35). Beaton et al. (36) claimed that sports involvement is present when individuals evaluate their participation in a sports activity that brings hedonic and symbolic value to their life. Studies conducted in recreational settings have shown that individuals who are more involved in an activity are more likely to remain customers (37, 38). Kyle et al. (39) noticed that the patterns of leisure involvement also depend on the nature of the activity and the participant's characteristics. Among senior people with a broad range of body mass indices, poor health and lack of interest are usually mentioned as constraints on PA (40–44). Many senior people also have a fear of falling and injury and feel insecure when exercising outdoors (45–48). Less is known about the perceived constraints on PA among obese senior people. No studies have examined whether or not perceived constraints can explain the increased risk of physical inactivity among obese senior people. Constraint captures the explanations for physical inactivity, which can be internal or external, intrapersonal or interpersonal, intervening or antecedent, blocking or inhibiting, and permanent or temporary (49). Based on the literature, this article has established three hypotheses, namely, (1) motivation for exercise positively affected WTP; (2) exercise involvement positively affected WTP; and (3) constraints negatively affected WTP. Our aim of this article is to examine the relationships among motivations, exercise involvement, constraints, and WTP in order to gain insights that will result in effective services to senior adult consumers in a sports and fitness center. This article is the first to discuss the factors influencing senior citizens' exercise consumption habits using WTP.

## Materials and methods

### Research method

This study is based on the concept of WTP, aiming to explore the factors influencing senior citizens' WTP more for exercise in the CSRCs outside of free hours. Pulido-Fernández and López-Sánchez (17) claimed that parametric methods are most used, but non-parametric approaches have some advantages with respect to the parametric analysis since the WTP estimators are easily assessed and are more robust to misspecifications of the probability distribution function. The structural equation model (SEM) has a few advantages over multiple regression and path analysis (50). Wertenbroch and Skiera (18) argued that the external validity of contingent valuation and related approaches may be limited. They provide less incentive to consumers to truthfully reveal their WTP because responses are hypothetical. Pulido-Fernández and López-Sánchez (17) asserted that there is still no standard about which is the appropriate statistical model to estimate the WTP. Hence, this article adopted the survey questionnaire to estimate WTP more from the study of Namkung and Jang (19).

### Sample design and data collection

In order to ensure the respondents who have visited a CSRC in Taiwan and whose age is above 65 years, this article adopted purposive sampling and the researchers waited for visitors beside the entrances of CSRCs during the free welfare time zones for seniors (8:00–10:00 a.m. during the whole week, and 2:00–4:00 p.m. from Monday to Friday). We provided both paper-based and mobile-based (with a QR code) questionnaires for participants. We collected 193 valid on-site questionnaires from the CSRCs. The last part of the demographic section of the questionnaire screened out invalid respondents. The valid collected data were analyzed using SEM.

### Measurement

This study adopted the mixed-method study including quantitative and qualitative analyses. After developing the questions from the well-established literature and then translating them into Mandarin Chinese to ensure participants' full comprehension, the first stage of this study invited five experts from the industry and academic fields in the sports industry to examine the validity of the questionnaire. The quantitative method in the second stage gathered data through the questionnaire. The questionnaire survey consisted of six sections: the first section introduced the purpose of this scale and informed participants that individual responses would be kept highly confidential and anonymous. The first section asked respondents to fill in their real feeling. The second section assessed a motivation construct made up of 15 items (Cronbach's α = 0.944). Examples of items include “Maintain physical health and physiological functions.” and “Keep pace with social trends;” an exercise involvement construct made up of 13 items (Cronbach's α = 0.950). Examples of items include “PA is very important to me;” and constraints made up of 13 items (Cronbach's α = 0.894). Examples of items include “I am more introverted” and “I never did PA before.” The participants were asked to provide their level of agreement to disagreement with the related statements, adopting a 5-point Likert scale from 1 for “highly disagree” to 5 for “highly agree.” This article puts all questionnaire items in Table 2.

**Table 2:** Major related extant research on WTP.

**Constructs**	**Items**	**Factor loading**	**CR**	**AVE**
Motivation	Q1 Maintain physical health and physiological functions	0.503	0.944	0.537
(The reasons to join PA)	Q2 Relax your mind and regulate your body and mind	0.519		
	Q3 Maintain physical strength and good spirits	0.492		
	Q4 keep a regular lifestyle	0.685		
	Q5 Make friends	0.774		
	Q6 Keep social contacts with peers	0.815		
	Q7 Become a member of the sport group and have a sense of belonging	0.864		
	Q8 Invite friends to join PA	0.662		
	Q9 Keep pace with social trends	0.812		
	Q10 Stay in touch with society and maintain social identity	0.862		
	Q11 Guide others	0.817		
	Q12 Challenge yourself	0.767		
	Q13 Unleash creativity	0.782		
	Q14 Learn new knowledge to enrich the quality of life	0.760		
	Q15 For self-realization	0.732		
Exercise Involvement (EI)	Q16 PA is very important to me	0.540	0.958	0.641
	Q17 Participating in PA is very satisfying	0.908		
	Q18 I am happy to join PA.	0.869		
	Q19 I am interested in joining PA.	0.936		
	Q20 Join PA is a pleasure	0.896		
	Q21 I love to join PA	0.822		
	Q22 PA accounts for a large portion in my life.	0.842		
	Q23 PA accounts for a central position in my life.	0.839		
	Q24 PA accounts for an important position in my life.	0.870		
	Q25 I love to discuss PA with my friends.	0.773		
	Q26 I can perform PA well.	0.690		
	Q27 I can explain a lot when other people play PA.	0.739		
	Q28 I like acting PA.	0.564		
Constraint (The reasons why I don't want to join PA)	Q29 I am more introverted	0.693	0.857	0.44
	Q30 PA requires special skills	0.580		
	Q31 I never did PA before	0.665		
	Q32 I feel uncomfortable doing PA	0.592		
	Q33 People who are important to me believe that I am not suitable to do PA.	0.636		
	Q34 People who are important to me can't accompany me to Join PA	0.672		
	Q35 I need to take care of grandchildren	0.594		
	Q36 I can't find the right person to go exercise with me	0.573		
	Q37 Bad weather	0.484		
	Q38 Insufficient safety of sports facilities	0.704		
	Q39 Too crowded	0.530		
	Q40 Poor sanitation	0.623		
	Q41 No relevant PA information	0.801		

Namkung and Jang (19) examined customers' WTP more for green practices in restaurants using hypothetical scenarios. Namkung and Jang (19) used one item (“How much more are you willing to pay for green practices at the restaurant?”) to measure consumers' WTP. This study adopts this previously validated questionnaire item from the study of Namkung and Jang (19). The third section addressed demographic variables and measured WTP using a Likert instrument (How much will you pay for exercise outside of the free welfare hours? 1: NT$0; 2: NT$1–5; 3: NT$6–10; 4: NT$11–15; 5: NT$16–20; 6: NT$21–25; 7: NT$26–30; 8: NT$31–35; 9: NT$36–40; 10: NT$41–45; 11: NT$46–50; 12: NT$51 and above; (US$1 = NT$28).

### Measurement model

This article used the purposive sampling method to collect on-site data from participants in 12 CSRCs over an 8-week period during the free welfare time zones for seniors (8:00–10:00 a.m. and 2:00–4:00 p.m.) in Taipei City.

One of the demographic questionnaire items asked respondents about their age range. If the respondent's answer was below 65 years old, their questionnaire would not be included in the further analysis. A total of 210 questionnaires were distributed, and a total of 193 valid samples were collected for analysis.

We examined the insignificant difference in WTP more between women and men using the *t*-test. We further examined the relationship between age group and WTP more using one-way analysis of variance (ANOVA) and the *post-hoc* Dunnett T3 test. This study further assessed the reliability, validity, and Spearman's correlation through SPSS version 26.0. Confirmatory factor analysis (CFA) and path analysis were undertaken with the IBM AMOS 26 Graphics.

## Results

### Descriptive statistics

Table 3 lists the descriptive statistics of the participant data. Of the 193 valid samples, 104 (53.9%) are men; 89 (46.1%) are women. A total of 56 (29%) respondents are aged 65–69 years; 52 (27%) respondents are aged 70–74 years; 28 (14.5%) respondents are aged 75–79 years; 43 (22.3%) respondents are aged 80–84 years; and there are 14 (7.3%) respondents above 85 years old.

**Table 3:** Demographics of the selected participants.

**Characteristics**	**Frequency (percentage %)**
**Gender**	
Male	104 (53.9%)
Female	89 (46.1%)
**Age**	
65–69	56 (29.0%)
70–74	52 (26.9%)
75–79	28 (14.5%)
80–84	43 (22.3%)
85 above	14 (7.3%)

We examined the insignificant difference in WTP more between women and men using the t-test. We further examined the relationship between age group and WTP more using one-way ANOVA. The Levene homogenous variance test value is 9.71 (p-value < 0.0001), indicating that the hypothesis of homogenous variance of WTP more is rejected. The *post-hoc* Dunnett T3 test with heterogeneous variance (Table 4) shows that WTP more in the groups of people aged 75–79, 70–74, and 65–69 years is higher than in the group of people aged 85 years and above. However, the WTP more of the group of people aged 80–84 years is only insignificantly higher than that of people in the group aged 85 years and above.

**Table 4:** WTP more comparison among five age groups.

**Age group**	***N***	**%**	**WTP more Mean**	**SD**	***F*-value**	***Post-hoc* Dunnett T3 test**
1.65–69	56	29%	8.52	4.15	5.834^**^	1 > 5^*^, 2 > 5^**^, 3 > 5^***^
2.70–74	52	27%	9.23	3.45		
3.75–79	28	15%	10.36	3.58		
4.80–84	43	22%	7.65	5.22		
5.85 and above	14	7%	4.14	5.16		
Total	193	100%	0.60	0.28		

WTP more: 1: NT$0; 2: NT$1–5; 3: NT$6–10; 4: NT$11–15; 5: NT$16–20; 6: NT$21–25; 7: NT$26–30; 8: NT$31–35; 9: NT$36–40; 10: NT$41–45; 11: NT$46–50; 12: NT$51 and above (US$1 = NT$28).

* p-value < 0.1;

** p-value < 0.05;

*** p-value < 0.01.

Since WTP more in the groups of participants aged 75–79, 70–74, and 65–69 years is higher than in the group of participants aged 85 years and above, the CSRC could motivate seniors aged 65–79 years, particularly for the 75–79 years age group, who are most willing to pay more, to experience sports with marketing promotions in order to raise their WTP for sports consumption outside of free welfare hours.

The average WTP more among people aged 65–69, 70–74, 75–79, 80–84, and 85 years and above is 8.52 (NT$31–40), 9.23 (NT$36–45), 10.36 (NT$41–50), 7.65 (NT$26–35), and 4.14 (NT$11–20), respectively. These empirical results indicated that the maximum average WTP more among different age groups is < NT$50.

### Reliability and validity

This study further assessed convergent validity and discriminant validity of all constructs through factor loadings, composite reliability (CR), and average variance extracted (AVE) in Table 2. All CR values show a greater degree of reliability than the recommended cutoff value of 0.70 and AVEs are >0.40, respectively (51).

### Bivariate correlations

Existing research has used the Spearman correlation analysis to indicate the associations between variables before SEM (50, 52). This article also used the Spearman correlation analysis to indicate significant links between research variables. This is illustrated in Table 5, which shows that motivation and exercise involvement are significantly positively related to WTP more. Constraints for exercise are insignificantly negatively related to WTP more.

**Table 5:** Bivariate correlations between research variables and descriptive statistics.

**Variables**	**Mean**	**SD**	**Motivation**	**Exercise involvement**	**Constraints**
WTP more	8.47	4.46	0.271^**^	0.217^**^	−0.074
Motivation	4.23	0.71	–	0.748^**^	−0.014
Exercise involvement	4.16	0.80	–	–	−0.013
Constraints	3.12	0.76			–

** Correlation is significant at 0.05 level.

### Confirmatory factor analysis and structural equation modeling

#### CFA validation

Confirmatory factor analysis is the first stage of the SEM that validates the goodness-of-fit of our model. The examination of the measurement model of the scales and the assessment of the path analysis were undertaken with the IBM AMOS 26 Graphics. The main criteria used to judge model fit, such as Bentler's (53) Comparative Fit Index (CFI) and Normal Fit Index (NFI), for which values >0.85 validate a good model fit (54), and the root mean square error of approximation (RMSEA), with a value of < 0.1 considered acceptable (55), are shown in Table 6. If the model does not fit well, modification indices (MIs) for the lambda matrix might be used to improve it (56).

**Table 6:** CFA for each construct and the research model.

**Construct**	**No of items**	**NFI**	**CFI**	**RMSEA**
Motivation	16–>5	0.96	0.98	0.09
Exercise involvement	12–>3	0.98	0.99	0.07
Constraint	15–>8	0.88	0.92	0.09
Overall model	21–>16	0.82	0.88	0.09

NFI, normal fit index; CFI, comparative fit index; RMSEA, root mean square error of approximation.

Table 6 shows that both each construct and the whole model achieve goodness-of-fit by eliminating unfit items through MI improvement (56). However, Shook et al. (57) advise that the Bollen–Stine bootstrapping approach can be used to adjust the model fit if the data cannot fulfill normal distribution. In particular, Thode (58) recommends the Shapiro–Wilk test as the best choice for normality testing of the dependent variable. This article used the Kolmogorov–Smirnov test with the Lilliefors correction and Shapiro–Wilk statistics for a normality test (57, 59). The *p*-values for these two normality tests are < 0.001, indicating that the data do not seem to fit a normal distribution. Hence, this article further used the Bollen–Stine bootstrapping method to modify the fitness indices.

#### Path analysis from the SEM model

The results of the SEM [χ^2^(115) = 315.85, p < 0.0001; NFI = 0.824; CFI = 0.879; RMSEA = 0.095] are shown in Table 7. However, due to the non-normal data in SEM, Shook et al. (57) suggest using the Bollen–Stine bootstrapping approach to adjust the model fit. The model fit indices in this study achieved a reasonable fit (51, 60) after Bollen–Stine bootstrapping, as shown in Table 7.

**Table 7:** Suggested goodness-of-fit statistics and acceptable cutoff criteria.

**Goodness-of-fit statistic**	**Goodness-of-fit statistics**	**After Bollen–Stine bootstrapping was employed**	**Suggested cut-off criteria**
NFI	0.82	0.99	>0.85
CFI	0.88	0.99	>0.85
RMSEA	0.09	0.03	<0.08

NFI, normal fit index; CFI, comparative fit index; RMSEA, root mean square error of approximation.

Table 8 indicates that motivation (β = 0.11^**^) has a significantly positive impact on WTP, supporting Hypothesis 1. Exercise involvement (β = 0.20^***^) has a significantly positive impact on WTP, supporting Hypothesis 2. In contrast, constraints have an insignificant effect on WTP, which does not support Hypothesis 3. Meanwhile, the coefficient of exercise involvement is larger than the coefficient of motivation, indicating that exercise involvement has a more positive impact on WTP.

**Table 8:** The result of SEM and hypothesis.

**Path**	**Standard estimate**	**S.E**.	**C ratio**	**p-value**
H1: WTP < – Motivation	0.11	0.657	2.139	0.032^**^
H2: WTP < – Exercise_Involvement	0.20	0.406	2.686	0.007^***^
H3: WTP < – Constraint	–0.07	0.396	−0.915	0.360

S.E., standard error; C ratio, critical ratio;

** Significant at level 0.05,

*** Significant level at level 0.01.

## Discussion

The findings of this article shed light on seniors' WTP processes in order to help develop effective marketing and exercise involvement strategies that drive future economic valuation decisions. Examining the relationship between age and WTP in Table 4, the *post-hoc* Dunnett T3 test shows that WTP among people aged 75–79, 70–74, and 65–69 years is higher than among people aged 85 years and older. However, the WTP of people aged 80–84 years is insignificantly higher than that of people aged 85 years and older. Hence, CSRCs could offer exercise courses for seniors aged 65–79 years, particularly for those aged 75–79 years, who are most willing to pay more, to create a win–win situation: one for the healthier seniors and the other for the wealthier CSRC.

The first aim of this study was to identify the determinants that could drive senior adults to participate in PA in the CSRC outside of the free welfare time zone. According to the empirical results, exercise involvement and motivation could drive seniors' WTP to do so. As for the second objective, this article examined whether motivation, exercise involvement, or constraints could inspire, or discourage senior adults to join PA in the CSRC. The empirical results suggest that both exercise involvement and motivation could have a positive impact on seniors' WTP; meanwhile, exercise involvement has a more significant effect on WTP. As long as the seniors recognized the importance and benefits of keeping healthy, they preferred to be active in various sports and fitness activities. This result is consistent with work from previous studies (61). Important preventative exercise has been linked to improved physical health (24, 25). Once a senior has regular exercise habit, they intend to pay more in order to maintain their health.

Meanwhile, seniors like to chat with their peers. One interesting topic is exercise. Buman et al. (61) indicated that social interaction is a crucial element in driving senior consumers to exercise involvement. The CSRC is a public area where everyone can not only perform some exercise but also connect with one another. If they are already used to doing regular exercise, they would spend more time on exercise or socializing outside of the free welfare hours, and will, therefore, pay more. According to our empirical findings, seniors would prefer to link with cutting-edge technology in modern society, such as social media chatting and wearable devices, while pursuing PA in the CSRC. Hence, launching advanced technology integrated with the exercise course would be a specific marketing strategy for the CSRC.

High body mass indices, poor health, and lack of interest are usually mentioned as constraints on PA (42, 45). However, this empirical result proves that as long as senior adults can develop their regular exercises, they are willing to pay more and exercise more often.

This article has several limitations. The first is the sample size. Due to the exploratory nature of this empirical work, our sample size was small because the participants had to be at least 65 years old. A second limitation is a generalizability. This article investigated only an empirical study in the CSRC in Taipei City. Thus, it may not be possible to generalize the findings to other private gyms and other sports and fitness facilities. The third limitation is convenience sampling, which excluded seniors who would not go to the CSRCs.

Wilson et al. (62) indicated that exercise could offer benefits in combating disease and improving the quality of life. Deci and Ryan (63) proposed the self-determination theory (SDT) and indicated that exercise can fulfill people's psychological needs, including competence (effectively facing and conquering challenging goals), autonomy (having a sense of ownership), and relatedness (keeping a close social connection with others). Our empirical study validates that exercise involvement (autonomy) and motivation (competence and relatedness) have a positive effect on WTP intention. These findings are also consistent with the theoretical perspective of SDT.

In this context, one of the strongest agreements in the motivation construct is “*I* could guide others.” This finding means that the seniors thought that they could pass their experiences onto similar-age or younger generations and by doing so, they help them strengthen their capacities and would obtain recognition from others (64). Hence, CSRCs in Taiwan could benchmark the practice of private gyms in other countries to recruit seniors as exercise coaches. Besides cultivating a patient, older coach, seniors would gain the peer learning effect that would provide more intention to experience exercise. The main policy implication of this study would be the promotion of regular exercise activities for seniors and the launching of exercise marketing campaigns in the community, linked to the local CSRC. Programs that promote social participation among seniors claim welfare in participants' happiness and a decrease in anxiety as well as depression levels (65, 66). The results of this study provide managers of CSRCs with the following decision-making recommendations: (1) initiate entry-level sports courses to cultivate in seniors the habit of exercise; (2) motivate seniors to participate in sports with marketing promotions in order to raise their WTP for sports consumption outside of free welfare hours; and (3) recruit seniors as exercise coaches or personal trainers to pass their experiences onto similar-age generations. In addition, the CSRCs could initiate resistance training, Tai Chi, and Eight Brocades exercise classes for seniors because these exercises have improved the pressure swing center and the stability limit for seniors (67–69).

## Conclusion

Willingness to pay in people aged 75–79, 70–74, and 65–69 years is higher than in those 85 years and above. CSRCs could offer exercise courses for seniors whose age is 65–79 years, particularly for those aged 75–79 years, who have the highest mean WTP more. Taking this paid course creates a win–win situation for healthier seniors and wealthier CSRC. The empirical results revealed that motivation and exercise involvement had a significantly positive impact on WTP intention, supporting hypotheses 1 and 2 through SEM. Future researchers could collaborate with colleagues in other countries to expand the sample size and sampling method and proceed with a cross-country study.

## Data availability statement

The raw data supporting the conclusions of this article will be made available by the authors, without undue reservation.

## Ethics statement

This study is extended from the project of sports centers (MOST 105-2410-H-003-048). This project was approved by the Research Ethics Committee, National Taiwan Normal University (REC Number: 201603HS010).

## Author contributions

C-YF contributed to conceptualization, literature review, methodology, software, and writing. P-YC contributed to literature review and data collection. YL contributed to writing. All authors contributed to the article and approved the submitted version.
